# Radiosensitivity in individuals with tuberous sclerosis complex

**DOI:** 10.1007/s12672-024-01395-1

**Published:** 2024-10-04

**Authors:** Lukas Kuhlmann, Jenny Stritzelberger, Rainer Fietkau, Luitpold V. Distel, Hajo M. Hamer

**Affiliations:** 1 Department of Radiation Oncology, Universitätsklinikum Erlangen, Friedrich-Alexander-Universität Erlangen-Nürnberg, Universitätsstraße 27, 91054 Erlangen, Germany; 2 Comprehensive Cancer Center Erlangen-EMN, 91054 Erlangen, Germany; 3 ERN EpiCARE, Epilepsy Center, Department of Neurology, Universitätsklinikum Erlangen, Friedrich-Alexander-Universität Erlangen-Nürnberg, Erlangen, Germany

**Keywords:** Tuberous sclerosis complex, Radiosensitivity, TSC1, TSC2, Ionizing radiation, Chromosomal aberrations, In situ hybridization

## Abstract

Benign tumors, but rarely cancer, are common in patients with tuberous sclerosis complex (TSC). Blood samples from patients undergoing treatment for TSC at our institution were analyzed for their individual sensitivity to ionizing radiation. Blood samples were collected from 13 adult patients with TSC. The samples were irradiated ex vivo and analyzed by 3-color fluorescence in situ hybridization. In each patient, aberrations were analyzed in 200 metaphases of chromosomes 1, 2, and 4 and scored as breaks. Radiosensitivity was determined by mean breaks per metaphase (B/M) and compared to both healthy donors and oncologic patients. The radiosensitivity (B/M) of the TSC patient cohort (n = 13; female: 46.2%, B/M: 0.48 ± 0.11) was clearly increased compared to healthy individuals of similar age (n = 90; female: 54.4%; B/M: 0.40 ± 0.09; p = 0.001). There was no difference compared to age-matched oncological patients (n = 78; female: 67.9%; B/M 0.49 ± 0.14; p = 0.246). Similarly, the proportion of radiosensitive (B/M > 0.5) and distinctly radiosensitive individuals (B/M > 0.6) was increased in the TSC and oncological patient cohorts (TSC: 30.8% and 7.7%, oncological patients: 46.2% and 14.1%) compared to the healthy individuals (11.1% and 2.2%). Although patients with TSC develop mostly benign and rarely malignant tumors, they are similarly sensitive to radiation as patients with malignant tumors.

**Supplementary Information:**

The online version contains supplementary material available at 10.1007/s12672-024-01395-1.

## Introduction

Tuberous sclerosis complex (TSC) is an autosomal dominant multisystem disorder [1, 2]. It is a rare disease with a prevalence of ~ 1: 6000 [3]. The disease is characterized by tumorigenesis and is associated with neurological and behavioral abnormalities [4]. One of the most common clinical manifestations of TSC is epilepsy, which occurs in approximately 80–90% of patients and results in high rates of drug-resistant epilepsy [5, 6]. De novo or inherited mutations in the TSC1 (9q34) or TSC2 (16p13.3) genes inactivate negative regulators of the mTOR tumor suppressor proteins hamartin and tuberin [7]. This leads to hyperactivation of the mTOR kinase pathway. Normally, in response to nutrient and growth factor stimulation, this pathway regulates cell growth and anabolic processes [8]. As a result, non-malignant tumors occur in many organs, including the brain, eyes, heart, kidney, skin, and lungs [3]. Accordingly, the clinical diagnostic criteria include cortical dysplasia, subependymal nodules, subependymal giant cell astrocytomas, cardiac rhabdomyomas, renal angiomyolipomas, retinal hamartomas, pulmonary lymphangioleiomyomatosis (LAM), and facial angiofibromas [4, 9–11]. TSC2 gene inactivation is associated with a more severe disease manifestation [12] and leads to a more severe epilepsy phenotype and neurological manifestations [13] than TSC1 gene inactivation. Additional patients with TSC2 mutations present symptoms at a younger age and have more severe symptoms, such as seizures, cortical tubera, and subependymal nodules [13].

Tumor predisposition syndromes are often associated with an increased risk of adverse therapeutic effects and secondary cancers due to increased tissue sensitivity when malignancies occur and radiation therapy is required [14, 15]. It is becoming increasingly clear that there are syndromes that mainly involve increased tissue sensitivity with no or only a slightly increased risk of tumor formation and secondary cancers [16]. An example of this is Cockayne’s syndrome with high tissue sensitivity and no increased risk of cancer [17]. Conversely, there are syndromes that are extremely susceptible to the development of tumors and secondary cancers induced by ionizing radiation, but are not otherwise associated with high tissue sensitivity [16]. One of these is Li-Fraumeni syndrome, with an already high risk of primary cancer of 50% by the age of 31 for female and 46 for male [18]. Secondary cancers are described in 30% of patients within 10.7 years after cancer treatment with ionizing radiation [19]. Most commonly, however, there is both increased susceptibility to tumors and increased tissue sensitivity. Examples are the classic radiosensitivity syndromes ataxia telangiectasia mutated and Nijmegen breakage syndrome. Both are characterized by an extremely high two to threefold increase in tissue sensitivity to ionizing radiation and a high risk of tumor development [20]. This suggests that an increased susceptibility to benign tumors, as in the case of TSC, could also imply an increased risk of tissue sensitivity.

Radiation sensitivity testing is not trivial. The goal is usually to determine tissue sensitivity prior to radiation therapy. It is very rare that tumor susceptibility testing is requested, which means testing to see if there is an increased risk of developing cancer or a secondary cancer due to cancer treatment. Chromosomal analysis of blood lymphocytes has proven useful in predicting radiation sensitivity [21, 22]. This has the advantage that blood is easy to obtain and chromosomal aberrations are a late endpoint where repair, signal transduction, cell cycle regulation and cell death control can be monitored. Chromosome analysis examines which chromosomal aberrations have occurred and scores them according to the number of underlying DNA double-strand breaks. The resulting value as breaks per metaphase then indicates how sensitive the patients are to ionizing radiation [23, 24]. Here we investigated whether patients with TSC have an increased susceptibility to ionizing radiation and studied this using chromosomal analysis.

## Materials and methods

Our cohort of patients with TSC consisted of 13 patients from whom we took a blood sample (NH4-Heparin, Sarstedt, Nürnbrecht, Germany). All patients with TSC were treated at our institution between May 2020 and August 2023. All patients who consented to the study were consecutively enrolled at the Epilepsy Center of the University Hospital Erlangen-Nuremberg. Healthy individuals from a cohort previously published [n = 208, mean age = 50.3 (SD = 17.7)] as well as oncological patients treated at the department of Radiation Oncology of the University Hospital Erlangen-Nuremberg [n = 347, mean age = 60.2 (SD = 13.0)] served as controls [24–26]. Individuals having any other medical condition or taking drugs suspected to increase radiation sensitivity like metformin, chloroquine, efavirenz, chloroquine, nelfinavir, vemurafenib, or immunotherapy were excluded from this study [27–31]. Additionally, we retrospectively analysed clinical records of all patients with TSC treated at our institution between 01/2003 and 08/2023 for the occurrence of benign and malignant tumours. This study was approved by the Ethics Committee of the Friedrich-Alexander-University Erlangen-Nuremberg (No. 21_19B). Written informed consent was obtained from all patients.

Three-color fluorescence in situ hybridization (FiSH) assay was performed to study radiation sensitivity. Blood from each participant was divided into two portions: one portion served as an non-irradiated control sample to detect the number of background aberrations, while the other portion received a 2 Gy dose using a 6 MV linear accelerator (Mevatron, Siemens, Germany). This dose of 2 Gy is equivalent to a single daily dose that is often given in a fractionated radiotherapy regimen. Blood-lymphocytes were stimulated with phytohemagglutinin (Biochrom, AG, Berlin, Germany) and cultured at 37 °C in an incubator. After 45 h of culture, the lymphocytes were arrested with colcemid (Gibco, Waltham, MS, USA) in the metaphase of the first cell division and chromosome preparation was performed. DNA hybridization with chromosome-specific probes was used to detect chromosomes 1, 2, and 4. Various fluorescent dyes were used for staining, and DAPI was used to counterstain the chromosomes. The entire procedure for three-color fluorescence in situ hybridization was performed as described previously [23, 25].

A fluorescence microscope (Zeiss, Axioplan 2, Göttingen, Germany) controlled by Metasystems software (Metafer 4 V3.10.1, Altlussheim, Germany) was used to automatically locate chromosome metaphase spreads at 100× magnification. A single image of each metaphase was captured at 630× magnification. Black and white images for each color (red, green, and blue) were obtained and used for evaluation of each metaphase spread [32]. Image analysis software (Biomas, Erlangen, Germany) was used to analyze a minimum of 200 metaphases. The assessment included scoring for translocations, dicentric chromosomes, acentric chromosomes, rings, deletions, insertions, and complex chromosomal rearrangements (CCRs). The data was then transferred to a spreadsheet (Excel, Microsoft Corporation, Redmond, WA, USA) and scores (breaks per metaphase, B/M) were calculated. Aberrations were scored based on the number of underlying chromosomal breaks according to the criteria of Savage and Simpson (33). The B/M score of the control sample was subtracted from the B/M score of the irradiated sample, to get the score for the 2 Gy dose.

SPSS Statistics 22.0 (IBM, Armonk, NY, USA) was used for statistical analysis. Two/One-tailed t-test with Welch’s correction and Mann–Whitney U-test were used to test for significant differences between groups, and p values < 0.05 were considered significant. GraphPad Prism (2020) was used for data visualization and statistical analysis. A lognormal distribution was chosen for curve fitting for all data sets, as suggested by Shapiro–Wilk/Kolmogorov–Smirnov test. The individual regression curves in relation to the data histogram are shown in supplementary Figure S1. The goodness of fit (in r2) was p = 0.915 for the TSC curve (Figure S1 A), p = 0.975 for the healthy curve (Figure S1 B), p = 0.956 for the oncological curve (Figure S1 C), p = 0.9818 for the young healthy curve (Figure S1 D) and p = 0.825 for the young oncological curve (Figure S1 E).

The following subgroups in the TSC cohort were compared: mutation: TSC1 (n = 3) to TSC2 (n = 4); age: younger than the median age of 37.6 years (n = 6) to older (n = 7); sex, female (n = 7) to male (n = 6);desease burden: less disease burden (n = 7) was defined as having fewer than four major diagnostic criteria [4] and more disease burden (n = 6) as having at least four criteria. Patients treated with the mTOR inhibitor Everolimus (n = 4) [33] were compared to patients not treated with this drug (n = 9).

## Results

### Patients and control groups

We evaluated radiation sensitivity in a total of 13 patients (female 46.2%, mean age 33 years ± 9, range 19–55) with TSC1 or TSC2. The genetics of seven patients were known, three had a mutation in the TSC1 gene and four in the TSC2 gene. We compared these patients to a cohort of 208 healthy individuals and a cohort of 347 oncological patients. The gender distribution was 46% female in TSC and approximately equal to 55% in the healthy cohort and 50% in the oncology cohort (Table 1). Since the average age of the two control groups was considerably higher than the age of the TSC patients, we created two sub cohorts by limiting the maximum age in these groups to 50 years, which left a cohort of 90 healthy individuals (average age 34 years), and a cohort of 78 oncological patients (average age 42 years). The oncology cohort included 53% of patients with rectal cancer, 33% with breast cancer, 7.5% with lung cancer and 6.5% with head and neck cancer.

**Table 1:** Comparison of sample size, mean age, gender distribution, cancer types and radiosensitivity (in B/M) of the analyzed cohorts: TSC patients, healthy individuals, young healthy individuals, oncological patients and young oncological patients

Cohort	TSC	Healthy	Healthy (< 50y)	Oncological	Oncological (< 50y)
Size	13	208	90	347	78
Mean age (SD)	33.0 (9.3)	50.3 (17.7)	33.9 (10.1)	60.2 (13.0)	42.3 (7.1)
Sex in % female	46	55	54	50	68
Cancer type (%)	Benign (100)	None	None	Breast (33) HNSCC (6.5) Lung (7.5) Rectal (53)	Breast (50) HNSCC (9) Lung (4) Rectal (37)
B/M 2 Gy (SD)	0.48 (0.11)	0.42 (0.1)	0.40 (0.09)	0.44 (0.13)	0.49 (0.14)
B/M 0 Gy SD)	0.006 (0.011)	0.025 (0.021)	0.019 (0.016)	0.056 (0.08)	0.056 (0.105)
Sensitive patients B/M > 0.5 (%)	4 (30.8)	37 (17.8)	10 (11.1)	96 (27.7)	36 (46.2)
Highly sensitive patients B/M > 0.6 (%)	1 (7.7)	10 (4.8)	2 (2.2)	35 (10.1)	11 (14.1)

Cohort with patients younger than 50 years = (< 50y); breast, patients with breast cancer; rectal, patients with rectal cancer; HNSCC, patients with head and neck squamous cell cancer; lung, patients with lung cancer; B/M breaks per metaphase

### Radiosensitivity in TSC compared to healthy individuals and patients suffering from cancer

**Fig. 1:**
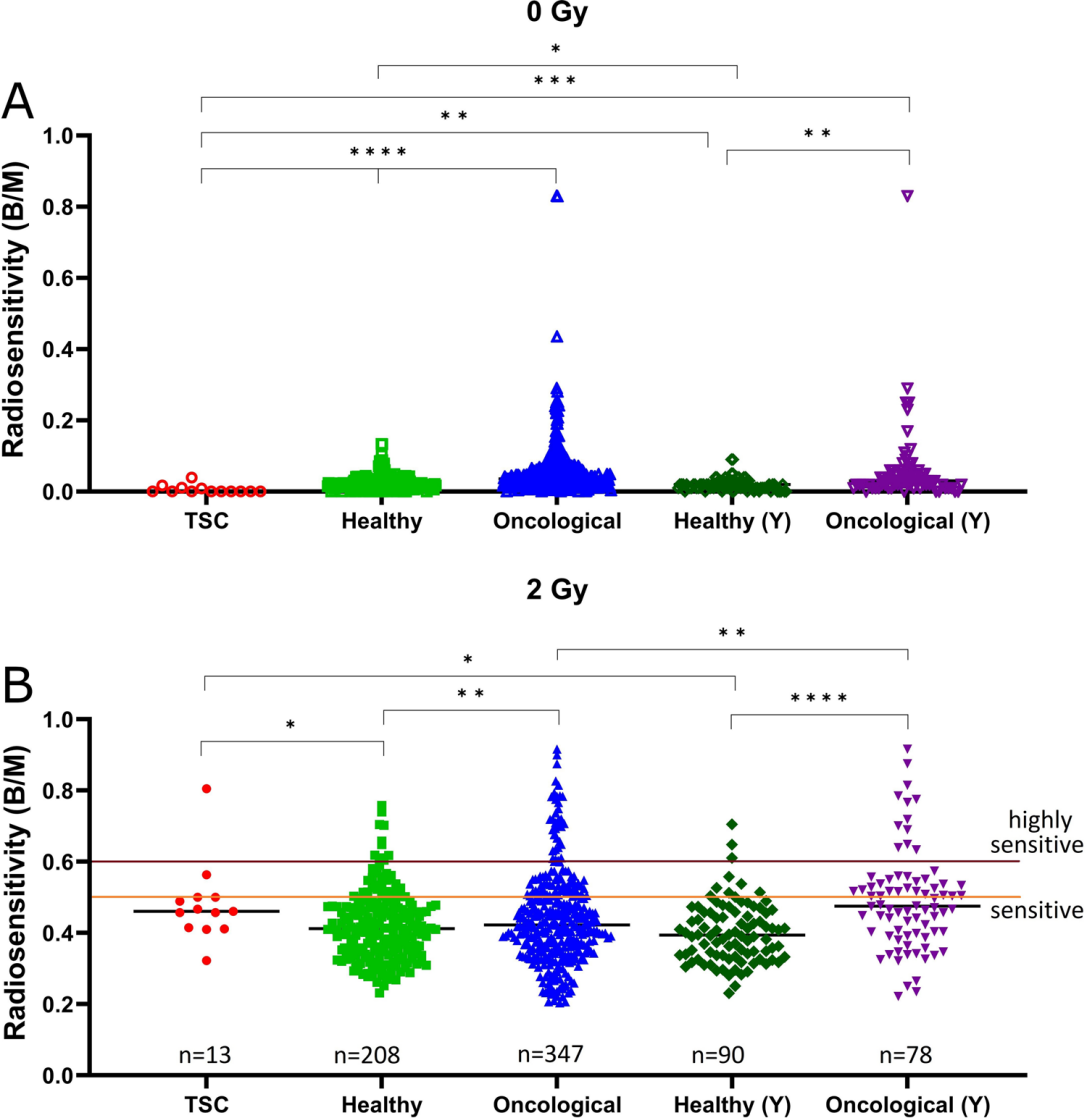
Radiation sensitivity in tuberous sclerosis patients compared to controls. Plots of individual scores in breaks per metaphase (B/M) for each cohort. Scores were determined by 3-color FISH chromosome aberration analysis. **A** B/M scores of untreated samples. **B** B/M scores after irradiation with 2 Gy and subtraction of the corresponding untreated background score. Horizontal lines indicate the thresholds for increased radiosensitivity (0.5 B/M) and   for highly increased radiosensitivity (0.6 B/M). *TSC* tuberous sclerosis; Y =  ≤ 50 years old; * = p ≤ 0.050, ** = p ≤ 0.010, *** = p ≤ 0.005, **** = p ≤ 0.001

The TSC cohort had lower background levels than healthy individuals and oncological patients (p < 0.001). This difference was less pronounced, but still significant in the younger cohorts (p = 0.001) for young healthy and p < 0.001 for young oncological individuals. The young healthy cohort contained significantly less background B/M than the total healthy cohort (p = 0.021). The difference between the young oncology cohort and the corresponding total cohort was not significant (p = 0.975, Fig. 1A). These values are used to subtract the background from the values obtained after irradiation. Since these background values show only a small absolute difference of 0.013 B/M for healthy and 0.05 B/M for oncological patients, the influence on the irradiated values is small.

After irradiation of patients’ blood with 2 Gy of ionizing radiation and after background subtraction, we observed chromosomal aberrations of an average of 0.48 breaks per metaphase (B/M) in the TSC cohort. This resulted in a clearly increased radiation sensitivity in this group compared to 0.42 B/M in healthy subjects (p = 0.038) and 0.40 B/M in young healthy subjects < 50 years of age (p = 0.014) (Table 1). In contrast, radiosensitivity in the oncology cohort and the young oncology cohort < 50 years was comparable to the TSC cohort (0.44 B/M, p = 0.246 and 0.49 B/M, p = 0.906, respectively). All cohorts had outliers on the upper side towards increased radiation sensitivity, while there were no outliers towards the lower side of radiation resistance (Fig. 1B).

The relative frequencies of B/M-Values were found to be log-normally distributed. Each cohort failed the Shapiro–Wilk test for normality (p = 0.006 for TSC, p < 0.001 healthy, p < 0.001 oncological, p = 0.007 young healthy and p = 0.002 young oncological). However, all cohorts except the oncological patients passed the Shapiro–Wilk test for log normality (p = 0.085 for TSC, p = 0.572 healthy, p = 0.028 oncological, p = 0.849 young healthy and p = 0.188 young oncological). The probability for log normal distribution as opposed to normal distribution was calculated to be 85.4% for TSC, 100% for healthy, 100% for oncological, 98.7% for young healthy and 97.7% for the young oncological cohort.

**Fig. 2:**
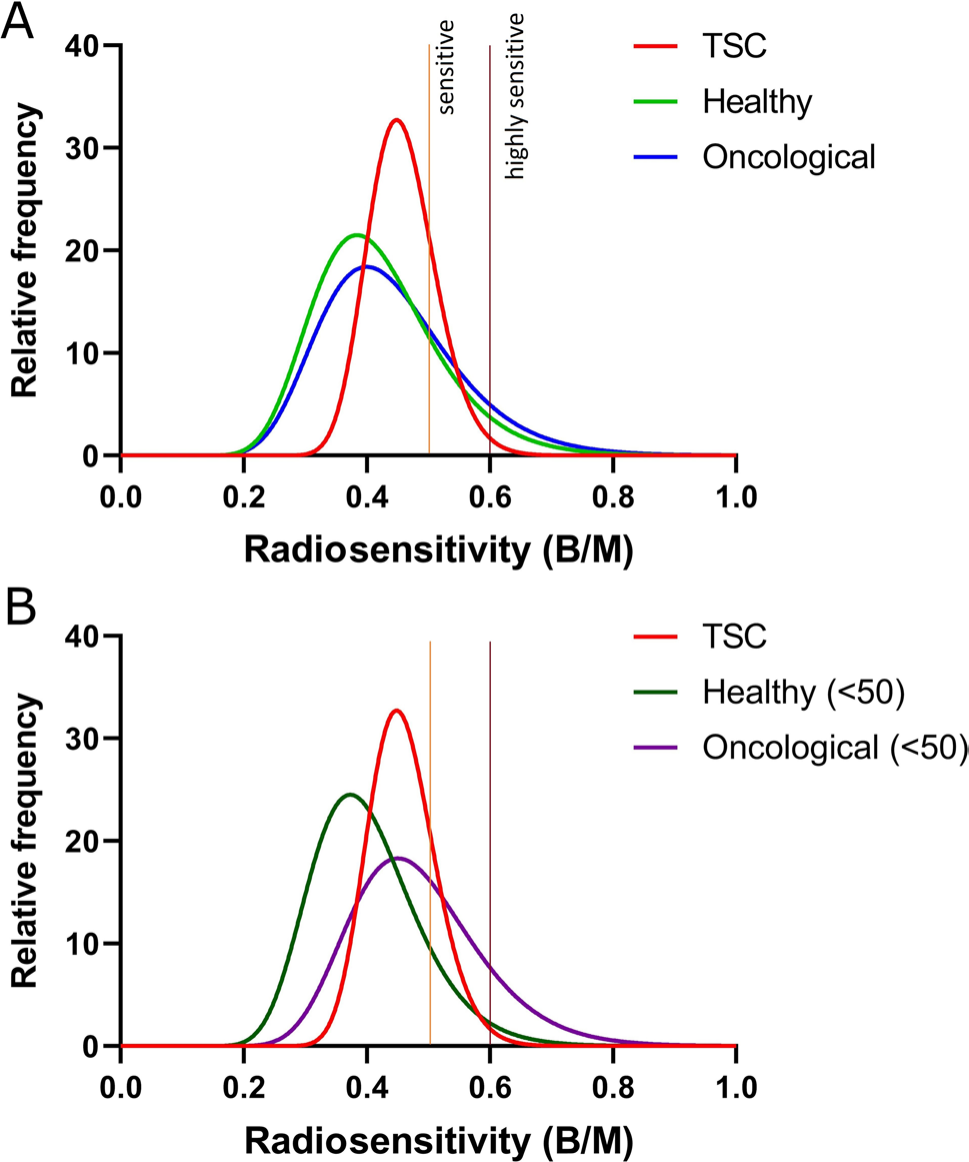
Distribution plots comparing radiosensitivity (B/M) between the TSC, healthy and oncological cohorts (**A**) and the TSC, young healthy and young oncological cohorts (**B**), respectively. The curves show a non-linear fit for lognormal distribution in all five data sets. Because lognormal curves are slightly asymmetrical, there are two different mean values to consider. The peak represents the most frequent radiosensitivity for a particular cohort, while the geometric mean represents the average radiosensitivity for that cohort. **A** TSC patients (red, peak: 0.448, geometric mean: 0.455), healthy individuals (green, peak: 0.384, geometric mean: 0.407) and oncological patients (blue, peak: 0.400, geometric mean: 0.426). **B** TSC patients (as above), Young healthy cohort (≤ 50 years) (dark green, peak: 0.373, geometric mean: 0.391) and young oncological patients (≤ 50 years) (purple, peak: 0.450, geometric mean: 0.472). Vertical lines indicate the thresholds for increased radiosensitivity (0.5 B/M) and for highly increased radiosensitivity (0.6 B/M)

A fitted curve over the frequency distribution shows the TSC cohort to be more frequently radiosensitive than all other cohorts (Fig. 2A, B). They were also less likely to be highly sensitive, especially compared to the young oncological cohort. This may however be due to the limited sample size of the TSC cohort, as the only highly sensitive TSC patient lay completely outside the curve (Supplementary Figure S1A). Other cohorts did not suffer the same problem (Supplementary Figure S1B–E). The most frequent B/M-value for TSC was higher than that of every other cohort except the young oncological cohort, which had a very similar peak (0.448 vs. 0.450 respectively).

### Proportion of radiosensitive individuals in different cohorts

In previous studies, we found that a value of 0.5 B/M or more can be considered an increased radiosensitivity, and a value of 0.6 B/M or more can be considered a clearly increased radiosensitivity [34]. The fraction of radiosensitive individuals (B/M > 0.5) significantly differed between the cohorts with 30.8% in TSC patients, 17.8% in healthy individuals, 11.1% in healthy individuals < 50y, 27.7% in oncological patients and 46.2% in oncological patients < 50y (Table 1). The fractions of highly radiosensitive individuals (B/M > 0.6) were 7.7% (TSC), 4.8% (healthy individuals), 10.1% (oncological patients), 2.2% (young healthy individuals) and 14.1% (young oncological patients).

### Difference in radiation sensitivity depending on clinical features

**Fig. 3:**
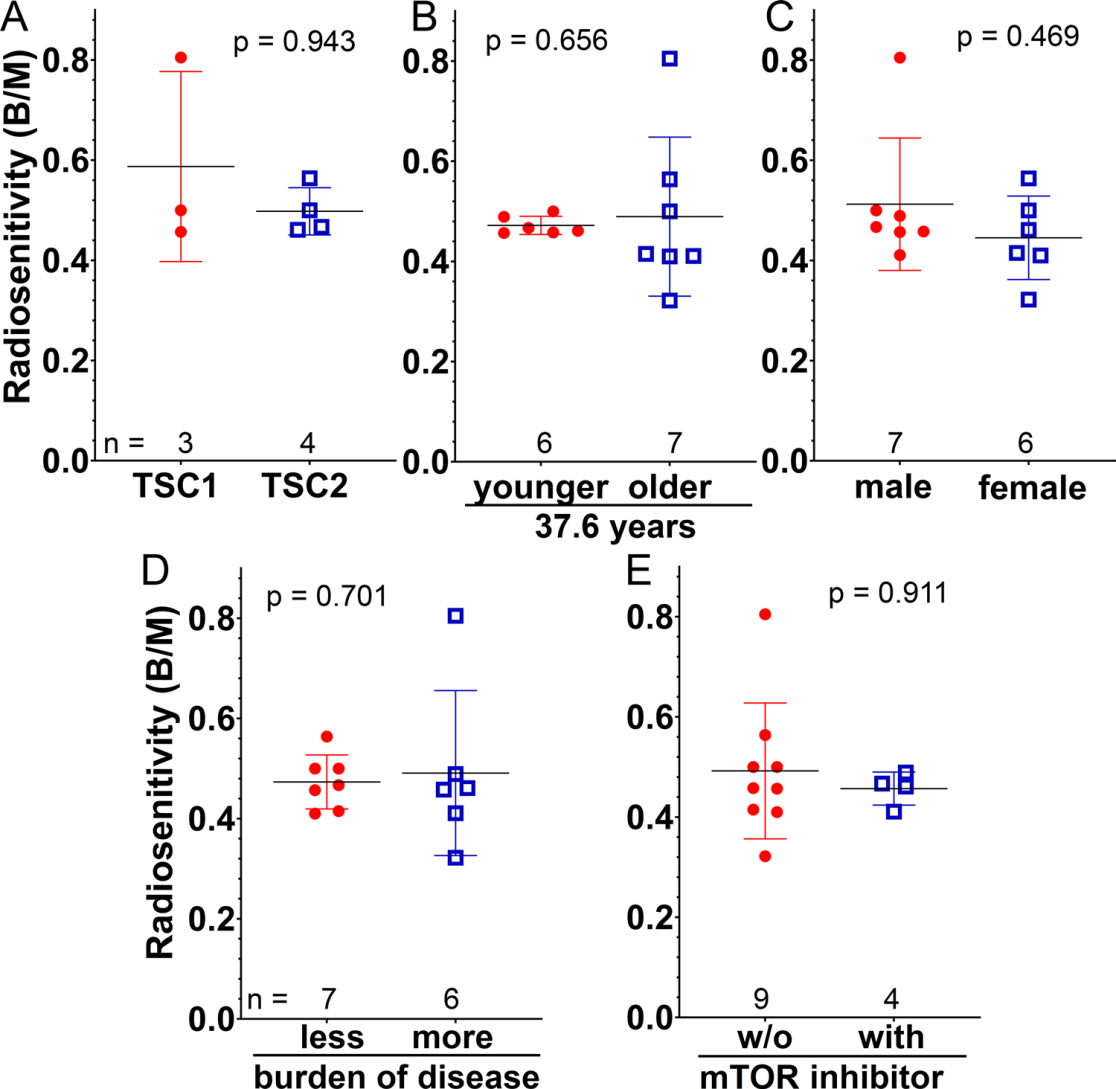
Comparison of various supgroups of the TSC cohort. The radiosensitivity of the following supgroups was compared: **A** TSC1 vs. TSC2, **B** patients younger than 37.6 years vs. older, **C** females vs. males, **D** patients with less disease burden (with less than four diagnostic criteria) vs. patients with more, **E** patients not treated with the mTOR inhibitor Everolimus vs. patients treated with the drug

We had data for three patients with a TSC1 mutation (mean B/M 0.57) and four patients with a TSC2 mutation (mean B/M 0.50), with missing genetic data for 6/13 patients. There was no distinct difference in radiation sensitivity between these two groups (p = 0.940), although the sample size was very small (Fig. 3A). However, it should be noted that the only outlier in the TSC cohort (B/M 0.8) had a TSC1 mutation. Additionally, we analyzed the influence of age (< / > 37.6 years, p = 0.656) and sex (p = 0.469) in the TSC cohort on radiosensitivity and found no significant differences (Fig. 3B, C). Similarly, we compared patients with less to more disease burden (p = 0.353) and patients using the mTOR inhibitor Everolimus to patients not using this drug (p = 0.911). Again, we found no difference between the groups (Fig. 3D, E).

### Clinical characteristics in the study cohort compared to a retrospective cohort

The sample of TSC patients was small with 13 individuals due to the relative rarity of the disease, therefore we performed a retrospective analysis to determine if the 13 patients studied were representative of all 38 patients previously treated at our hospital during the last 20 years. We studied both clinical TSC manifestations and medication (Table 2). We compared the following four groups: all patients (n = 38, consisting of the 13 patients tested and 25 patients in whom we retrospectively analyzed medical records), all tested TSC patients (n=13), the TSC patients with average radiation sensitivity (n = 9), and the TSC patients with increased radiation sensitivity (n = 4). All patients suffered from epilepsy, as this was the reason for their treatment in our clinic. The frequency of drug-resistant epilepsy as defined by ILAE-criteria was comparable in all groups (p = 0.901). Tubera were present in all patients. Radiosensitive TSC patients had a similar disease burden compared to the average sensitive patients (3.0 vs 3.2 major diagnostic criteria, p = 0.89). The retrospective cohort had less disease burden (2.3) compared to the radiosensitive (p = 0.05) and average (p = 0.03) cohorts. However, these results may be subject to bias due to the retrospective nature of the analysis, as symptoms may have been under-reported in the older, retrospective cohort and the 13 patients tested are currently being treated and followed up in our clinics. A mean of 2.3 medications was used by the retrospective cohort, 2.8 medications by the average and 2.0 medications by the highly radiosensitive patients (Table 2). The usage of antiseizure medication (ASM) was roughly equally distributed among all 38 and the 13 studied patients with levetiracetam being the most frequently prescribed ASM. Valproate was taken by 6 of the patients in the entire and 1 patient in the study cohort. Notably, only one patient (1/38, 2.6%) had a malignant tumor (papillary thyroid carcinoma and hepatocellular carcinoma). This patient was included in the retrospective analysis and was not tested for radiosensitivity.

**Table 2:** Clinical characteristics of all 38 patients with TSC, andthe 13 tested patients with TSC

Parameter	Variable	Cohort		Radiosensitivity	
		All n = 38	Tested n = 13	Average B/M n = 9	Increased B/M n = 4
	Mean age (SD)	38.2 (10.6)	33.0 (9.3)	36.1 (11.1)	36.8 (4.3)
	Sex females n (%)	15 (39.5)	7 (53.8)	5 (55.6)	2 (50)
Mutation	TSC1/2/unknown	8/8/22	3/4/6	1/2/6	2/2/0

Clinical manifestation					
Location	Symptom (%)				
Cerebral	Epilepsy	38 (100)	13 (100)	9 (100.0)	4 (100.0)
	Drug-resistant epilepsy	22 (57.9)	0 (0)	6 (66.6)	2 (50.0)
	Tubera	38 (100)	13 (100)	9 (100.0)	4 (100.0)
	Hamartomas	3 (7.9)	3 (23.1)	3 (33.3)	0 (0)
	Giant cell astrocytoma	11 (28.9)	4 (30.8)	2 (22.2)	0 (0)
	Subependymal nodules	7 (18.4)	2 (15.4)	1 (11.1)	1 (25)
Cardiac	Rhabdomyomas	4 (10.5)	3 (23.1)	3 (33.3)	1 (25)
Cutaneous	Angiofibromas	5 (13.2)	4 (30.8)	3 (33.3)	1 (25)
	Fibromas	6 (15.8)	0 (0)	0 (0)	2 (50)
	Angiokeratomas	1 (2.6)	1 (7.7)	1 (11.1)	0 (0)
	Adenoma sebacea	1 (2.6)	1 (7.7)	0 (0)	0 (0)
Pulmonary	Lymphangioleiomyomatosis	1 (2.6)	1 (7.7)	1 (11.1)	2 (50)
Renal	Angiomyolipomas	13 (34.2)	10 (76.9)	7 (77.8)	1 (25)
	Kidney cysts	2 (5.3)	1 (7.7)	1 (11.1)	0 (0)
Average number of tumors		0.93	2.2	2.3	2.0

Medication					
Drug (category)	Drug (%)				
ASM	Brivaracetam	1 (2.6)	0 (0)	0 (0)	0 (0)
	Carbamazepin	5 (13.2)	0 (0)	0 (0)	0 (0)
	Lacosamid	7 (18.4)	0 (0)	0 (0)	0 (0)
	Lamotrigin	9 (23.7)	4 (30,8)	2 (22.2)	2 (50)
	Levetiracetam	15 (39.5)	7 (53,8)	6 (66.7)	1 (25)
	Topiramat	6 (15.8)	3 (23,1)	2 (22.2)	1 (25)
	Oxcarbazepin	6 (15.8)	1 (7,7)	1 (11.1)	0 (0)
	Perampanel	4 (10.5)	4 (30,8)	2 (22.2)	2 (50)
	Phenobarbital	1 (2.6)	1 (7,7)	1 (11.1)	0 (0)
	Phenytoin	2 (5.3)	1 (7,7)	1 (11.1)	0 (0)
	Primidon	2 (5.3)	1 (7,7)	1 (11.1)	0 (0)
	Felbamat	1 (2.6)	0 (0)	0 (0)	0 (0)
	Valproat	6 (15.8)	1 (7,7)	1 (11.1)	0 (0)
	Vimpat	1 (2.6)	0 (0)	0 (0)	0 (0)
	Zonisamid	1 (2.6)	1 (7,7)	1 (11.1)	0 (0)
mTOR inhibitor	Everolimus	4 (10.5)	4 (30,8)	4 (44.4)	0 (0)
Statine	Fluvastatin	1 (2.6)	1 (7,7)	1 (11.1)	0 (0)
	Simvastatin	1 (2.6)	0 (0)	0 (0)	0 (0)
	Ramipril	2 (5.3)	0 (0)	0 (0)	0 (0)
	Torem	1 (2.6)	0 (0)	0 (0)	0 (0)
	L-Thyrox	3 (7.9)	2 (15,4)	1 (11.1)	1 (25)
	Pantoprazol	1 (2.6)	0 (0)	0 (0)	0 (0)
	Sertralin	0 (0)	0 (0)	0 (0)	0 (0)
	Tramadol	1 (2.6)	0 (0)	0 (0)	0 (0)
	Allopurinol	1 (26)	0 (0)	0 (0)	0 (0)
	ASS	1 (2.6)	0 (0)	0 (0)	0 (0)
	Folic acid	2 (5.3)	2 (15,4)	1 (11.1)	1 (25)
Average number of drugs/patient		2.3	2.5	2.8	2.0

The cohort of the tested TSC patients was additionally  split into two subgroups (average radiosensitivity, B/M < 0.5, n = 9, and increased radiosensitivity, B/M ≥ 0.5, n = 4). All groups were compared in regard to mean age, gender distribution, mutation type (TSC1 or TSC2), symptoms and medication

## Discussion

On average, the 13 TSC patients in our study had an increased sensitivity to ionizing radiation that was comparable to that of patients with malignant tumors in our study. In contrast, almost all except for one of the 38 TSC patients treated at our hospital had tumors that were exclusively benign. An obvious comparison group were healthy individuals. Compared to them, TSC patients were significantly more sensitive to ionizing radiation.

Age is the only factor in patient characteristics that clearly influences radiation sensitivity [25], apart from the underlying diseases that defined the groups. Therefore, we matched the cohorts only in this respect. Since most tumors are unlikely to occur in younger patients unless they have a genetic predisposition, the age restriction and matching process had a greater impact on the radiation sensitivity of the oncological patients than on the healthy individuals. This explains why the young oncological cohort was more sensitive to ionizing radiation than the unmatched oncological cohort of older patients. The young healthy cohort was slightly, but not significantly, less sensitive to radiation than the general healthy cohort, which we have previously observed [25]. In this study, age had little effect on radiosensitivity, although it is generally accepted that repair capacity decreases [35] and radiosensitivity increases with age [25]. In addition to age, there are often gender differences. However, there are no gender differences in radiation sensitivity [26]. We compared female with male TSC patients and did not find any difference (p = 0.469).

The severity of the manifestations of TSC varies greatly from one individual to another and even from one identical twin to another. Differences in the mutations that occur in TSC1 versus TSC2 and other poorly defined factors are likely responsible for this phenotypic heterogeneity [36]. Similarly, differences among the mutation spectrum and the genetic expression could lead to a difference in radiosensitivity. It is possible that patients with a higher burden of disease may have an increased sensitivity to radiation. We studied therefore whether the radiosensitive patients suffered more from the disease and had a higher disease burden. In the four radiosensitive TSC patients, however, we did not find a higher disease burden and patients with higher disease burden had no increased radiosensitivity. Mutations in TSC2 lead to a more severe form of the disease in multiple organs and to a higher disease burden [12]. Therefore, we also investigated whether TSC2 individuals are more sensitive to radiation. Due to the small number of known mutations in our cohort and the fact that the outlier patient had a mutation in TSC1, we could not find a difference between the radiosensitivity of TSC1 and TSC2 individuals.

For a long time, only symptomatic therapies were available for patients with TSC. With the discovery of the pathogenesis of TSC as a negative regulator of the mTOR complex 1 and the corresponding therapy with mTOR inhibitors, specific treatment for the cause of the disease became possible for the first time. The question is whether treatment with an mTOR inhibitor could also influence the radiosensitivity.

Everolimus was less frequently used in the entire cohort (10.5%) because it is a relatively new drug and the 13 included patients were therefore more likely to receive it than the retrospective, older cohort (30.8%). We found no difference in radiosensitivity between the nine patients without the drug versus the four patients using the drug (p = 0.911), indicating that the mTOR inhibitor probably did not affect radiosensitivity in TSC patients in this study. However, the four patients with increased radiosensitivity were not taking the drug. Another drug suspected to increase radiosensitivity is valproic acid [31]. Of the 13 patients, only one patient with normal radiosensitivity (0.415 B/M) was taking valproic acid and was therefore not considered relevant for our analysis. Nevertheless, it is conceivable that the radiation sensitivity of patients who are already more sensitive due to TSC could be further increased by Valproate. The ability of the other drugs to influence radiosensitivity is not known.

Molecularly, the TSC complex consisting of the proteins TSC1, TSC2 and TBC1D7 functions as an inhibitor to the mTOR complex 1 (mTORC1) by deactivating Rheb, which is needed to activate mTORC1. mTORC1 controls a number of metabolic functions and inhibits apoptosis [37]. Loss of TSC1/2 function causes mTORC1 to be constitutively active in the cells of TSC patients. This makes the cells insensitive to most growth-suppressing signals [38]. In TSC competent cells, p53 responds to DNA damage by activating AMPK, which phosphorylates TSC2. This leads to a decrease in mTORC1 and therefore to a reduction of the cells metabolism and an increase in apoptosis rate [39]. The increased radiosensitivity in patients with TSC mutations could be explained by the reduced ability of cells to respond to DNA damage in this way. Without TSC, mTORC1 is not inhibited and damaged cells do not undergo apoptosis. However, TSC function is incompletely understood and one of the most highly integrated signaling nodes found in all cells. Its ability to perceive and relay cell intrinsic and extrinsic signals is key to the control of cell, tissue and organismal homeostasis and growth. There are other mechanisms that could lead to the increased radiosensitivity. One mechanism, indicated by Ferlazzo, is that phosphorylation and therefore activation of the repair protein ATM occurs delayed. Simultaneously radiation induced a higher number of MRE11 foci, which also indicates a problem with repair [40] An impairment of nucleoshuttling of the ATM protein to the nucleus and a delayed phosphorylation of ATM has been described in several diseases with neurological defects and cancer proneness [41] e.g. Xeroderma Pigmentosum [42], Huntington’s disease [43] and Neurofibromatosis type 1 [44]. Another interesting aspect is that the TSC1 gene product hamartin is bound to the outer membrane of mitochondria and is involved in the regulation of apoptosis by binding to HSP70 [45]. The TSC2 gene product tuberin is also associated with mitochondria. Mitochondrial regulation of these proteins may play an important role in mTOR activation and thus cell growth control [46].

There are two implications for patients with increased radiosensitivity. One is that increased radiosensitivity is usually associated with increased susceptibility to cancer. The consequence is that patients undergoing X-ray diagnostics should be diagnosed with modern equipment that produces only low doses of imaging radiation [20]. In addition, dose reduction algorithms should be used.

The second are the deterministic risks associated with interventional diagnostics and radiotherapy. Radiotherapy, with its high doses of about 2 Gy per fraction and total doses of up to 76 Gy, has been made possible by powerful equipment that make adverse effects of radiotherapy rare. However, increased radiosensitivity increases the likelihood of treatment-related side effects in the individual patient [14, 15, 47]. In those cases, it is possible to reduce the dose to reduce the risk of side effects. A large portion (30.8%) of the TSC patients in our study were at or above the threshold of 0.5 B/M, where we define increased radiosensitivity. We recommend reducing the fraction dose for radiotherapy of malignant tumors for individuals with B/M values above 0.55. Therefore, if a malignant tumor was present and was to be treated with radiotherapy in our cohort, a dose reduction would only be recommended for two patients. For the patient with 0.59 B/M, we would recommend a dose reduction of 5–10% of the fraction dose. For the patient with the extreme radiation sensitivity of 0.81 B/M, we would recommend a reduction in the fraction dose of between 25–40%. We recommend reducing the fraction dose and keeping the number of fractions constant because we argue that for a patient with a correspondingly reduced dose, the effect is the same as for a healthy patient in whom ionizing radiation has a normal effect. Of course, there is a degree of uncertainty in testing radiation sensitivity, which is a clear limitation. Therefore, our dose reduction is very careful to avoid underdosing and the occurrence of relapse.

One of the unanswered questions about TSC is whether patients have an increased risk of malignant cancer in addition to the extremely high risk of developing benign tumors. Data on increased cancer rates with TSC are scarce. Renal cell carcinomas appear to be increased and the incidence of cancer to occur at younger ages [48]. Conversely, low levels of TSC1 and TSC2 are associated with an unfavorable prognosis in breast cancer and some other cancers [49]. In our retrospective analysis, one patient (1/38, 2.6%) was suffering from a malignant tumor. Interestingly, this patient’s age (64 years) was considerably higher than the mean age of our cohort, leaving the question open whether the carcinomas could be attributed to potentially increased radiosensitivity due to the underlying TSC disease or other risk factors.

The small number of patients due to the rarity of the disease is a clear limitation of this study. Nevertheless, our study shows that TSC patients have an increased radiation sensitivity comparable to patients with malignant tumors. In addition, there are probably individual outliers with significantly increased radiation sensitivity.

## Conclusions

Patients with TSC have a slightly increased risk of increased sensitivity to ionizing radiation. There may be a few individuals with TSC who are at high risk. Our findings are independent of clinical features such as age, sex or disease severity, however, our sample size was small, making cautious interpretation of our results necessary.

## Supplementary information

Supplementary Material 1: Figure S1. Overlay of Histogram and fitted curve for sensitivity scores) against their relative frequency

## Data Availability

The datasets generated and/or analyzed in the present study are not publicly accessible, but can be provided by the corresponding author upon reasonable request.

## Declarations

### Guidelines and regulations

All methods were carried out in accordance with relevant guidelines and regulations.
