# Supplementary material for: Radiosensitivity in individuals with tuberous sclerosis complex
Source: Discov Oncol. 2024 Oct 4;15:525. doi: 10.1007/s12672-024-01395-1 (PMC11452609; doi:10.1007/s12672-024-01395-1)

**Supplementary Figure 1:** Overlay of Histogram and fitted curve for sensitivity scores (in B/M, Bin= 0.05)) against their relative frequency.


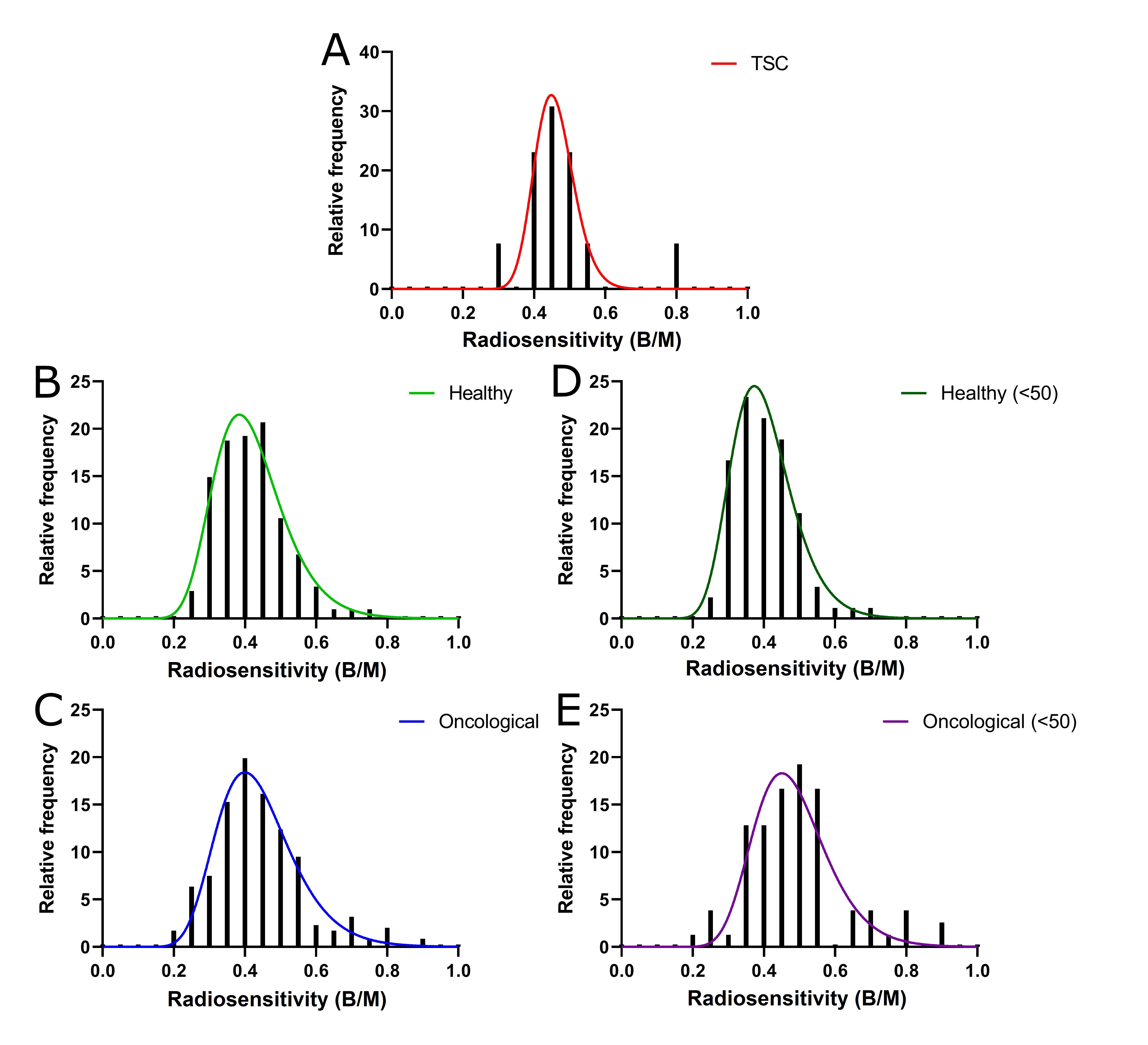

Supplement: Supplementary file 1 — Supplementary Material 1: Figure S1. Overlay of Histogram and fitted curve for sensitivity scores) against their relative frequency [file 12672_2024_1395_MOESM1_ESM.docx]
